# Lymphoedema screening: setting the standard

**DOI:** 10.1038/s41416-020-0848-0

**Published:** 2020-05-04

**Authors:** Cheryl L. Brunelle, Alphonse G. Taghian

**Affiliations:** 1 Department of Physical and Occupational Therapy, Massachusetts General Hospital, Boston, MA 02114 USA; 2 Department of Radiation Oncology, Massachusetts General Hospital, Boston, MA 02114 USA

**Keywords:** Breast cancer, Breast cancer

## Abstract

Existing literature which is changing practice should be scrutinised, in the interest of all women at risk for lymphoedema after breast cancer (BC). Bundred et al.’s prospective, multicentre trial of 1100 women made several solid findings, and novel screening recommendations presented may assist in incorporating lymphoedema screening into standard of care.

## Main

Screening for lymphoedema after breast cancer (BC) surgery is recommended^1–5^ but not standard of care currently. There is no universal diagnostic criteria for BC-related lymphoedema (BCRL); criteria are often correlated but not interchangeable,^6^ hindering research progress.^7^ The recent trend towards screening for subclinical BCRL with bioimpedance spectroscopy (BIS) and treating at a lower threshold lacks evidence. Resources for BCRL screening and treatment are scarce and quality of life (QOL) is impacted by BCRL. Therefore, this movement should be scrutinised, and Bundred et al.^8^ in the current issue of the *British Journal of Cancer* have provided the needed data to make evidence-based recommendations with this study.

The authors conducted a multicentre prospective study of 1100 patients comparing multi-frequency BIS with arm volume measurement. The aims of the study included determining which test has better accuracy, identifying factors predicting BCRL, and understanding its effect on QOL. Patients were measured with perometry (arm volume) and BIS at baseline and throughout 5-year follow-up. BCRL was defined as a relative arm volume increase (RAVI) ≥ 10% or BIS L-Dex increase two or three standard deviations from baseline (>7.5 or >10 respectively). Patients with RAVI ≥ 10%, swelling in the lower arm or hand, or BCRL symptoms were diagnosed with BCRL and fitted for compression.

Of note, Bundred et al.^8^ found that 8.3% of patients had volume differences ≥200 mL between arms at baseline. This is consistent with Sun et al.,^9^ where 2.9% and 28.3% of patients had arm volume differences at baseline of 10% and 5%, respectively. This study highlights the importance of baseline measurements, echoing the finding that not incorporating baseline measures results in up to 50% misdiagnosis.^9^ Preoperative baseline measurement should be the standard.^1–5^

This study found a 2-year BCRL incidence of 22.4% via RAVI, and 45.2% and 57.6% via BIS (l-Dex increase ≥10 and 7.5, respectively).^8^ While they note that there is no gold standard definition of BCRL, they use perometry as benchmark in this study. In the established literature, 2-year incidence of BCRL in patients undergoing axillary clearance is not as high as 57.6% (via BIS in Bundred et al.^8^). A highly cited meta-analysis found that the 2-year incidence from 18 studies of patients with axillary dissection was 19.9% (range 13.5–28.2%),^10^ consistent with the 22.4% incidence via RAVI ≥ 10% in this paper.

Studies have found that BIS is responsible for significant false positives,^11^ and in another study of 58 patients diagnosed with BCRL via ICG lymphography (the true gold standard), 21 had a normal l-Dex via BIS—a 36% false negative rate.^12^ Bundred et al.^8^ aptly point out that the literature base touting BIS’s ability to detect subclinical BCRL is limited—studies lack baseline measurements and long-term follow-up to identify patients who return to a normal after an abnormal l-Dex reading without intervention.^13^ BIS is not established to identify subclinical BCRL, and from Bundred et al.’s study^8^ on 1100 patients, RAVI ≥ 10% is recommended for screening.

Although RAVI 5% to <10% is a risk factor for progression to RAVI ≥ 10%,^14^ Bundred et al.^8^ correctly state that the threshold for early treatment with compression is not evidence based. Stout et al.’s observational study,^15^ which treated RAVI ≥ 3% with a sleeve for 4 weeks, found arm volume reductions of 4.1%, which were maintained over 4.8 months. This was a small study (*n* = 43) without a control group; arm volume may have decreased without intervention. Further, Specht et al. found that only 18.7% of patients with an RVC of 5% to <10% occurring >3 months after surgery progressed to RVC ≥ 10%.^14^ More research is needed before start treating BCRL at RAVI 3% or 5%.

We applaud Bundred et al.’s addition to the literature around risk factors and QOL.^8^ They had several interesting findings, for example that BMI at surgery predicted reduced QOL and progression of BCRL even after sleeve fitting. Improved education and resources are needed. They found that a high percentage of patients with BCRL report swelling or heaviness, which supports many national organisation’s recommendation^1–5^ of incorporating symptoms into BCRL screening.

Bundred et al.^8^ found that after compression sleeve application, patient QOL scores increased for patients with RAVI ≥ 5% but not for those with RAVI < 5%. This is one of the first studies showing that patients may find solace in treating BCRL early. One other study found improvements in depression and anxiety following treatment for BCRL.^16^ This may result from increased patient sense of autonomy in management of a highly feared sequelae of BC treatment.

Finally, Bundred et al.^8^ summarised screening recommendations based on their novel scoring system for BCRL progression risk: risk increased as scores increased. A small percentage (12%) of patients with a low risk score at one month progressed to RAVI ≥ 10%. In contrast, 76.7% of patients with a high-risk score progressed. Bundred et al. aptly observe that the risk of progression in the low risk group cannot be ignored, recommending allotment of resources towards high-risk populations whilst educating lower risk groups to ensure self-referral with any new symptoms.^8^ If resources allow, we recommend screening all patients; 65 patients in the low risk group in this study progressed to BCRL.

In conclusion, we applaud Bundred et al.’s well-conducted and important study.^8^ This study was unable to demonstrate any evidence supporting BIS for detection of subclinical BCRL. Baseline and longitudinal arm volume measures and symptoms monitoring is imperative for screening. The effectiveness of early intervention with a sleeve at RAVI 5% to prevent progression to RAVI ≥ 10% has not been established. We add that BCRL screening is important to detect clinical BCRL in its early stages (RAVI ≥ 10%), not just subclinical BCRL. Although recommended, it has unfortunately not been implemented as standard of care.

This trial has many strengths that need to be considered. In the interest of all women at risk for lymphoedema after BC, we must scrutinise the literature which is changing practice for its limitations. Future directions include controlled, long-term studies that delineate the ability of BIS to identify subclinical BCRL and identify the true threshold for early treatment. The standard needs to be set, but it must be based on a strong research foundation.

## Data Availability

Not applicable (editorial).
